# Comparative Transcriptomics Unveil the Crucial Genes Involved in Coumarin Biosynthesis in *Peucedanum praeruptorum* Dunn

**DOI:** 10.3389/fpls.2022.899819

**Published:** 2022-05-17

**Authors:** Cheng Song, Xiaoli Li, Bin Jia, Li Liu, Peipei Wei, Muhammad Aamir Manzoor, Fang Wang, Biqi Yao Li, Guanglin Wang, Cunwu Chen, Bangxing Han

**Affiliations:** ^1^College of Biological and Pharmaceutical Engineering, West Anhui University, Lu’an, China; ^2^Anhui Engineering Laboratory for Conservation and Sustainable Utilization of Traditional Chinese Medicine Resources, Lu’an, China; ^3^College of Life Science, Anhui Agricultural University, Hefei, China; ^4^Analytical and Testing Center, West Anhui University, Lu’an, China

**Keywords:** bolting, lignification, coumarin biosynthesis, transcriptional regulation, ABC transporters

## Abstract

*Peucedanum praeruptorum* Dunn is a commonly used traditional Chinese medicine that is abundant in furano- and dihydropyrano coumarins. When *P. praeruptorum* reaches the bolting stage, the roots gradually lignified, and the content of coumarins declines rapidly. Non-bolting has always been a decisive factor for harvesting the *P. praeruptorum* materials. To evaluate the amount of coumarin components in unbolted and bolted *P. praeruptorum*, the variations of praeruptorin A, praeruptorin B, praeruptorin E, peucedanocoumarin I, and peucedanocoumarin II were determined. Additionally, 336,505 transcripts were obtained from the comparative transcriptome data. Among them, a total of 1,573 differentially expressed genes were screened out. To identify the critical genes involved in coumarin biosynthesis, comparative transcriptomics coupled with co-expression associated analysis was conducted. Finally, coumarin biosynthesis-related eighteen candidate genes were selected for the validation of *q*PCR. Additionally, a phylogenetic tree and the expression profile of ATP-binding cassette (ABC) transporters were constructed. To clarify the main genes in the regulation of coumarin biosynthesis, the interaction network of the co-expression genes from thirteen modules was constructed. Current results exhibited the significant increment of praeruptorin A, praeruptorin B and praeruptorin E in the bolted *P. praeruptorum*. Although, peucedanocoumarin I and peucedanocoumarin II were slightly increased. Besides the content of coumarins, the essential genes involved in the coumarin biosynthesis also exhibited an overall downward trend after bolting. Three *peroxidases* (*PRXs*) involved in the production of lignin monomers had been demonstrated to be downregulated. *PAL*, *C4H*, *HCT*, *COMT*, *CCoAOMT*, and some ABC transporters were dramatically downregulated at the bolting stage. These results indicated that the downregulation of coumarin biosynthetic genes in the bolted *P. praeruptorum* ultimately reduced the formation of coumarins. However, the mechanism through which bolting indirectly affects the formation of coumarin still needs extra functional verification.

## Introduction

*Peucedanum praeruptorum* is a perennial herb in the Umbelliferae family whose dried roots are commonly used in traditional Chinese medicine (Song et al., 2021). Coumarins, which are rich in *P. praeruptorum*, have a wide range of applications in the prevention and treatment of cardiovascular and cerebrovascular diseases, anti-inflammatory, reversal of multidrug resistance, anti-cancer, and neuroprotection (Lee et al., 2015; Stelzhammer et al., 2017; Wang et al., 2017; Liu et al., 2020). The medicinal ingredients of *P. praeruptorum* are mainly furan- and dihydropyran-type coumarins (Zhao et al., 2015; Yu et al., 2020). Furanocoumarins are a class of secondary metabolites derived from structurally simple coumarins (Dugrand-Judek et al., 2015). Structurally, furanocoumarins are available in two isomeric forms: linear and angular, which are considered to originate from the phenylpropanoid pathway (Tian et al., 2017). The upstream genes of the phenylpropanoid pathway were involved in the formation of coumarins (Sui et al., 2019). PAL is the first rate-limiting enzyme in the regulation of coumarin biosynthesis, whose expression was susceptible to multiple abiotic stresses (Shang et al., 2012; Sui et al., 2019). 4CL catalyzed the formation of cinnamoyl-CoA, *p*-coumaryl-CoA, caffeoyl-CoA, ferulic-acid-CoA, and various hydroxycinnamate-CoA esters (Rastogi et al., 2013; Li and Nair, 2015). From three *4CL*s found in *P. praeruptorum*, *Pp4CL1* preferentially takes coumarate and ferulic acid as substrates, although it can also use caffeic acid, cinnamic acid, *o*-coumaric acid, and other precursors. *Pp4CL7/4CL10* lacked catalytic activity for hydroxycinnamic acid (Liu et al., 2017). The ortho-hydroxylation of hydroxycinnamate is an essential step in the biosynthesis of coumarins, especially for the subsequent cyclization of coumarin lactones (Matsumoto et al., 2012). C2′H is required for the formation of umbelliferone. The expression of *C2′H* was high in the roots of *P. praeruptorum* and was induced by MeJA and UV-B treatments (Yao et al., 2017). *Caffeic acid O-methyltransferase-similar* (*COMT-S*) was found to be responsible for the O-methylation of hydroxycoumarins (Zhao et al., 2019). Cinnamic acid was catalyzed and lactonized by cinnamic acid-4-hydroxylase (C4H), 4-coumaric acid-CoA ligase (4CL), and p-coumaroyl-CoA 2′-hydroxylase (C2′H) to yield umbelliferone (Song et al., 2020). Some CYP450 family members and MDR transporters may participate in the biosynthesis and transportation of coumarins (Zhao et al., 2015). However, the downstream branch and specific coumarin transporters related to dihydropyranocoumarin remain unclear.

The biosynthesis of coumarins still requires some post-modifying enzymes, which are mainly composed of methyltransferase, o-methyltransferase, prenyltransferase, and monooxygenase. Umbelliferone dimethylallyl transferase (UDT) plays an important role in the prenylation of umbelliferone (Munakata et al., 2016). Umbelliferone can be prenylated at the C6 and C8 positions to yield linear and angular furocoumarins, respectively. Psoralen is hydroxylated at the C5 and C8 positions to form xanthotoxol and bergaptol, respectively. The osthenol catalyzed by U8DT satisfies the structural basis for dihydropyranocoumarins. Depending on U6DT, umbelliferone is prenylated to form demethylsuberosin (DMS). Marmesin synthase (MS) catalyzes DMS to form marmesin, which is then converted to psoralen via psoralen synthase (PS) (Jian et al., 2020). Marmesin is hydroxylated by marmesin monooxygenase (MO) to produce bergaptol or xanthol. Bergaptol O-methyltransferase (BMT) is involved in the O-methylation reaction of bergaptol with high substrate specificity (Zhao et al., 2016b). Imperatorin is produced from bergaptol and xanthomol by prenyltransferase (PT), bergaptol-O-methyltransferase (BMT), and xanthomol-O-methyltransferase (XMT) to form imperatorin, isoimperatorin, and other furanocoumarins (Munakata et al., 2020). A portion of the osthenol precursors are added to angelicin, while the residue will form dihydrofuranocoumarins by multi-step reactions (Munakata et al., 2016). Based on the biogenic pathway, lomatin may participate in the biosynthesis of praeruptorin A, praeruptorin B, praeruptorin E, and other dihydrofuranocoumarins through PT, OMT, and CYP450 monooxygenase. The current issue is that the key genes or transcription factors responsible for the biosynthesis and regulation of *P. praeruptorum* coumarins have not been widely identified and investigated. To some extent, this limits the application of synthetic biology strategies for large-scale production of such active ingredients in heterologous expression systems.

Early bolting has a significant impact on the accumulation of secondary metabolites in traditional Chinese medicine (Zhou et al., 2014). The bolted herbs in the Umbelliferae family, such as *Peucedanum*, *Angelica*, *Saposhnikovia*, *Notopterygium*, and *Glehnia*, were generally not harvested (Yu et al., 2019; Song et al., 2021). *P. praeruptorum* began to lignify once it entered into reproductive growth, and the content of coumarins gradually declined (Guo et al., 2021; Li et al., 2021). During the reproductive growth, a large amount of nutrients are consumed. A lack of carbon sources led to an increment in the secondary xylem area and a decrease in coumarins (Chen et al., 2019; Robe et al., 2021). Our previous study suggested that post-transcriptional modification, signal transduction, and secondary metabolism might play vital roles in coumarin biosynthesis (Song et al., 2021). Among them, the key enzymes of the phenylpropanoid pathway such as ATP-binding cassette (ABC) transporters, apoptosis-related genes, and circadian rhythm-related genes participated in the regulation of coumarin biosynthesis. Despite that, what is the expression pattern of key genes involved in coumarin biosynthesis between the bolted and unbolted *P. praeruptorum*, and which ABC transporter subfamily may be involved in coumarin transportation and distribution? How do the co-expressed genes involved in coumarin biosynthesis connect and interact? These are still unanswered questions that are worthy of further exploration. Here, the contents of five pyranocoumarins were determined at the bolting stages. The lignification of *P. praeruptorum* root was determined by phloroglucinol staining. A total of 18 candidate genes were identified, which are involved in the regulation of coumarin biosynthesis. Among them, *PAL*, *C3H*, *COMT*, and *HCT* in the phenylpropanoid pathway in bolted *P. praeruptorum* and *AS*, *PS1*, and *BMT* involved in coumarin biosynthesis were dramatically downregulated Three *PRXs* related to lignin polymerization were also negatively regulated. These findings will contribute to a better understanding of the coumarin biosynthetic pathway and bolting mechanism in *P. praeruptorum.*

## Materials and Methods

### Samples and Reagents

The samples for the experiment were taken from the Ta-pieh Mountain Medicinal Botanical Garden of West Anhui University in October 2020. All *P. praeruptorum* samples were grown for more than one year. The whole plants of the unbolted and bolted *P. praeruptorum* were collected for further experiments. Ten biological replicates were taken at the bolting stages for the determination of coumarins, and three biological replicates were taken for *q*PCR analysis. By comparing the original plant to the reference medicinal material (Batch No. WKQ-DZYC-01607) from Sichuan Vikeqi Biotechnology Co., Ltd. (Chengdu, China), the authenticity of the plant was confirmed. The fibrous roots of *P. praeruptorum* were removed. The taproots were preserved and rinsed with sterile water, and the surface water was absorbed and dried naturally.

Peucedanocoumarin I (Shanghai Yuanye Biotechnology Co., Ltd., batch number: B50414, purity > 95.0%). Praeruptorin A (National Institute for Food and Drug Control, purity > 99.4%). Peucedanocoumarin II (Shanghai Yuanye Biotechnology Co., Ltd., batch number: B5041, purity > 97.8%). Praeruptorin B (National Institute for Food and Drug Control, purity > 98.9%). Praeruptorin E (Shanghai Yuanye Biotechnology Co., Ltd., batch number: B20036, purity > 99.9%). Methanol (GR, Shanghai McLean Biochemical Technology Co., Ltd.). Other reagents were of analytical grade.

### The Histochemical Staining

*Peucedanum praeruptorum* taproots were stained with phloroglucinol-hydrochloric acid dye solution by the previous methods (Liljegren, 2010; Chauhan et al., 2015). In brief, 1mL of concentrated hydrochloric acid was dropped on the cross-section of the front beard and left for five minutes. Then, 1mL of the phloroglucinol-alcohol mixture was dropped to dye the lignified cell wall. Finally, the dyed pink area in the xylem was recorded.

### Preparation of Coumarin Extraction

The extraction of total coumarin was referred to as the Chinese Pharmacopoeia, followed by the previous method (Hou et al., 2010). The roots of *P. praeruptorum* were naturally dried in the shade and then pulverized into a coarse powder. 0.5 g of powder was mixed with 25 mL of chloroform and ultrasonically extracted for 10 minutes (250 W, 33 kHz). After cooling the extract, the lost weight was replaced with chloroform. To prepare the test solution, 5 mL of the continuous filtrate was evaporated to dryness and dissolved with proper methanol. All samples were filtered through a 0.45 μm organic-based microporous membrane (ANPEL Laboratory Technologies (Shanghai) Inc.) prior to the HPLC analysis.

### Optimization of HPLC Conditions and Methodology

The content of coumarins was determined using LC-2030C high-performance liquid chromatography (Shimadzu, Japan). The chromatographic column used in the test was ZORBAX Eclipse Plus C18 (150 mm x 4.6 mm, 5 μm) (Agilent, United States). Methanol-water (volume ratio of 75:25) was used as the mobile phase. The flow rate was set at 1.0 mL/min. The column temperature was set at 30°C, and the detection wavelength was at 235 nm. The injection volume was 10 μL. To produce the reference substance solutions, the appropriate amounts of the peucedanocoumarin I, praeruptorin A, peucedanocoumarin II, praeruptorin B, and praeruptorin E standards were mixed with methanol, respectively. The concentrations of Peucedanocoumarin I, praeruptorin A, peucedanocoumarin II, praeruptorin B, and praeruptorin E were 30.59 g/mL, 79.94 g/mL, 29.82 g/mL, 86.88 g/mL, and 58.69 g/mL, respectively.

The methodological investigation concluded with the precision test, stability test, repeatability test, and recovery rate test by using previous methods (Tao et al., 2009). The precision test was conducted by injecting 10 μL of test solution under the specified chromatographic conditions and repeating six times. The stability test included the injection of the test solution at 4, 8, 12, 16, 20, and 24 h intervals under the specified chromatographic conditions. The repeatability test evaluates six batches of *P. praeruptorum*. The test solution was injected at a volume of 10 μL under the specified chromatographic conditions. The recovery rate experiment was conducted to weigh 0.50 g of *P. praeruptorum* (including 0.49 mg/g of peucedanocoumarin I, 6.04 mg/g of praeruptorin A, 0.46 mg/g of peucedanocoumarin II, 3.43 mg/g of praeruptorin B, and 2.09 mg/g of praeruptorin E). The test was repeated six times. Coumarin standards were added to each batch, and the peak area of each sample was measured. The recovery rate and relative standard deviation (RSD) of each standard were calculated, respectively. The optimized chromatography method was used to determine the five coumarins in the samples, and ten biological replicates were performed for each coumarin.

### The Hierarchical Clustering, Phylogenetic Tree and Expression Profile of the Differential Genes

The raw data of the transcriptome used for the analysis of expression profiles were obtained from the Sequence Read Archive (SRA) database^1^. The BioProject accession was PRJNA714368. To investigate the differential genes at the bolting stages, transcripts from the annual bolted and unbolted *P. praeruptorum* were selected for subsequent analysis. CDD, KOG, COG, NR, NT, PFAM, Swissprot, and TrEMBL databases were used for the functional annotation (Altschul et al., 1997). The GO function annotation is obtained based on the annotation of the transcript from Swissprot and TrEMBL. The KEGG Automatic Annotation Server (KAAS) was used to obtain KEGG annotation information (Moriya et al., 2007). The transdecoder software^2^ was used for CDS prediction after blasting the transcripts with databases. Based on the annotation information, all possible unigenes were screened and the blastn (*e*-value < 10^–5^) was used for additional validation. By setting the significant difference (*p* < 0.01), false discovery rate (FDR) correction (*q* < 0.05), and | fold change| > 2, a total of 1,573 genes with significantly differential expression were obtained (Benjamini and Hochberg, 1995; Anders and Huber, 2010).

The abundance of transcripts directly reflects the level of expression of a specific gene. In the experiment, the TPM value was used to compare the gene expression between the two groups. Subsequently, the salmon tool was used to calculate the expression levels by using RNA-seq data (Patro et al., 2017). For the replicates in the same group, the expression level is the average of all repeated data. The TPM values of all the unigenes were used for hierarchical clustering and expression profiling of differentially expressed genes. TBtools (*v.*1.098) was used to compare the gene expression profiles among groups (Chen et al., 2020). MEGA (*v*.6.0.6) was used to align the target sequences and construct a phylogenetic tree based on the Neighbor-Joining method (Tamura et al., 2013).

### Analysis of Coumarin Biosynthesis Genes by Quantitative Real-Time PCR Analysis

Before performing the total RNA extraction, the sampling equipment was disinfected and de-RNAsed. Fresh *P. praeruptorum* roots were collected, and the surface was quickly cleaned with RNase-free water. The samples were placed in an enzyme-free tube before being quickly frozen in liquid nitrogen. After it had completely frozen, it was transferred to a refrigerator set at –80 degrees Celsius for storage. Each *P. praeruptorum* sample was ground to 100 mg in liquid nitrogen. Total RNA was isolated from bolted and unbolted *P. praeruptorum* using the UNIQ-10 column Trizol total RNA extraction kit (Sangon Biotech Ltd., Shanghai). Electrophoresis was used to detect RNA concentration. Subsequently, mRNA was isolated and fragmented. The mixture was centrifuged for 3-5 s after it had been mixed. After 10 min of incubation at 25°C, the reaction was performed at 50°C for 30 min and at 85°C for 5 min to obtain cDNA. *GAPDH* was selected as a reference gene referred to in this study (Zhao et al., 2016a). Based on the sequences of eighteen candidate genes and the reference gene, Primer Premier 5.0 was used to design the primer sequences (Supplementary Table 1). The *q*PCR experiment was carried out using the StepOne Plus real-time PCR equipment (ABI, Foster, CA, United States). Relative quantification of target genes was performed by using the SYBR Green I method with 2X SG Fast *q*PCR Master Mix (High Rox, B639273, BBI, ABI). The 2^–ΔΔ^ ^Ct^ method was applied to calculate the relative expression of the target genes after bolting (Livak and Schmittgen, 2001).

### The Interaction Network Analysis

Several genes relevant to coumarin biosynthesis were screened out from 13 gene modules of the WGCNA data. Briefly, the WGCNA script was used to create a gene set matrix for the co-expression correlation analysis (Langfelder and Horvath, 2008). The differential expression profile obtained by the transcriptome analysis was used in the WGCNA. Following the selection of an appropriate soft threshold, the co-expressed gene modules were performed to determine the number of genes in each module. The co-expression correlation coefficient between genes was calculated first based on the measured gene expression levels, and then the genes were clustered using euclidean distance by drawing a gene tree. The phenotypic traits were weighted, and the correlation and credibility of each gene module were calculated in relation to them. The core module was selected based on its relevance and significance. Based on the weighted scores of these gene pairs, an intergenic interaction network was visualized through the Cytoscape (*v.*3.9.0) (Doncheva et al., 2019).

## Results and Discussion

### Sample Collection and Histochemical Staining of *P. praeruptorum* Roots

**FIGURE 1:**
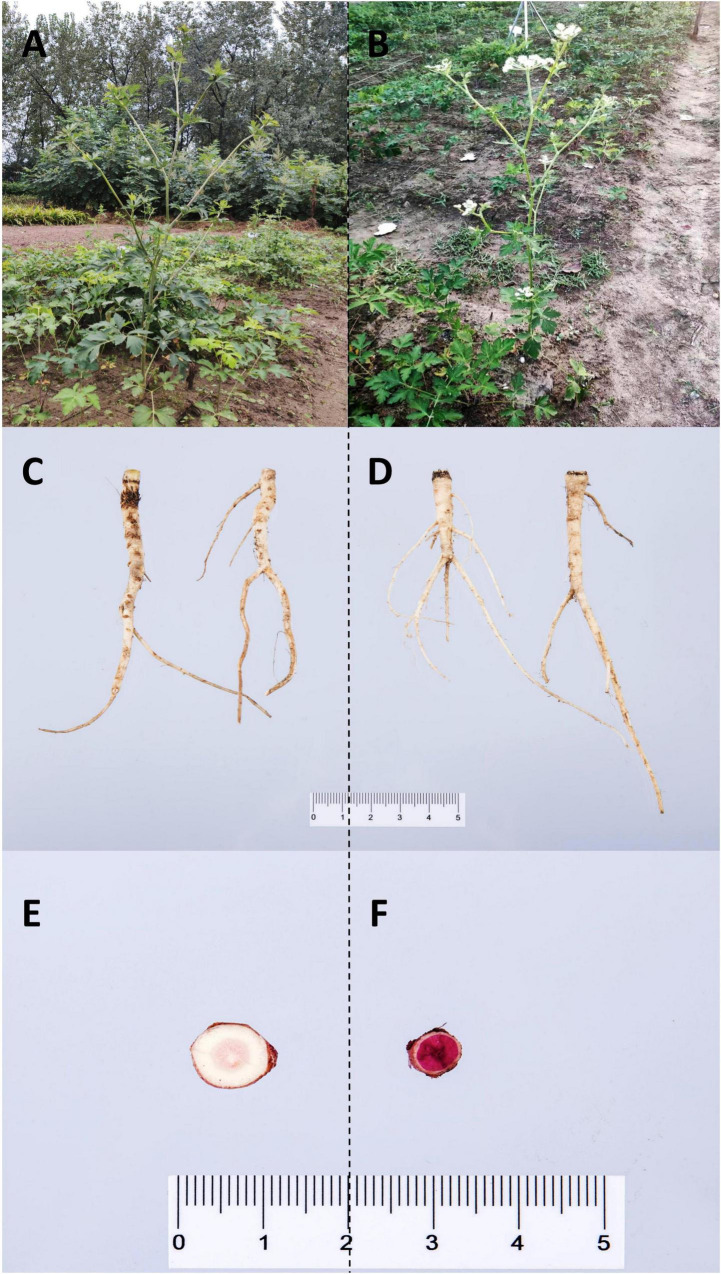
Sample collection and the phloroglucinol staining of unbolted and bolted *P. praeruptorum* roots. **(A)** Unbolted *P. praeruptorum*, **(B)** Bolted *P. praeruptorum*, **(C)** The root of unbolted *P. praeruptorum*, **(D)** The root of bolted *P. praeruptorum*, **(E)** Phloroglucinol staining of the cross section of the unbolted taproot, **(F)** Phloroglucinol staining of the cross section of the bolted taproot.

Due to the influence of genetic, ecological, and growth circumstances, *P. praeruptorum* from the same period may grow at different rates (Liang et al., 2018). To compare the degree of the lignification at the bolting stage, both unbolted and bolted *P. praeruptorum* roots were collected (Figure 1). In the bolting stage, the taproot of *P. praeruptorum* is relatively slender and has more fibrous roots. The results from phloroglucinol staining showed that the area of bolted xylem was darker and larger, which implied that the lignin content in the bolted roots was higher (Chen et al., 2019). Early bolting has become a crucial factor affecting the quality of the crude materials. The quality of these materials depends on when they are harvested (Zhao et al., 2011; Li et al., 2020). Currently, there is no conclusive evidence that bolting results in a loss of ability to use medications (Pouteau and Albertini, 2009; Li et al., 2022). The bolted *P. praeruptorum* conveys large amounts of nutrients to the aerial parts, which may be the possible reason for early bolting (Song et al., 2021).

### Extraction of Coumarins and Methodology Investigation

**FIGURE 2:**
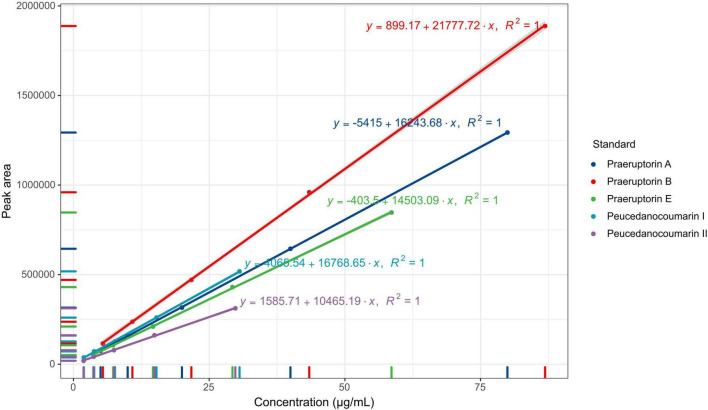
The standard curve and equations of the five coumarin standards.

The five coumarins (peucedanocoumarin I, praeruptorin A, peucedanocoumarin II, praeruptorin B, and praeruptorin E) were determined from the methanol extraction of *P. praeruptorum* roots. The chromatographic conditions were investigated as a priority to optimize the detection of the samples. These results indicated that the five coumarins kept a better linear relationship in the range of concentration (Figure 2). The linear ranges of peucedanocoumarin I, praeruptorin A, peucedanocoumarin II, praeruptorin B, and praeruptorin E were 0.48∼7.65 μg, 1.25∼19.99 μg, 0.47∼7.46 μg, 1.36∼21.72 μg, and 0.92∼14.65 μg, respectively. Six independent injections were performed under the optimized chromatographic conditions. The RSD of the peak areas of peucedanocoumarin I, praeruptorin A, peucedanocoumarin II, praeruptorin B, and praeruptorin E were 0.3, 0.26, 0.27, 0.26, and 0.25%, respectively, which indicated that the precision of the instrument was good. The samples were injected at 4, 8, 12, 16, 20, and 24 h, with the RSDs of the peak areas of 0.97, 0.18, 0.18, 0.06, and 0.26%. Six samples from the same batch were tested, and the RSDs of the five coumarin contents were 3.62, 0.92, 2.68, 2.49, and 1.43%, implying good repeatability. The recoveries of peucedanocoumarin I, praeruptorin A, peucedanocoumarin II, praeruptorin B, and praeruptorin E were 99.97, 99.79, 101.17, 99.73, and 99.95%, respectively. The RSDs of the five standards were 0.28, 0.17, 0.93, 0.23, 0.21%, respectively, which suggested the test method has good accuracy.

### Determination of the Coumarins in the Unbolted and Bolted *P. praeruptorum*

**FIGURE 3:**
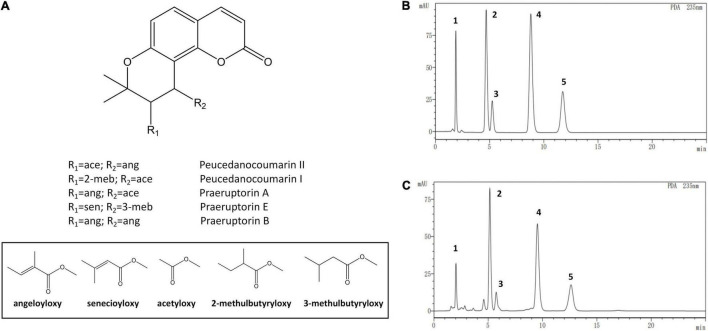
The chemical structure and high performance liquid chromatograms of five coumarin components. **(A)** The structures of the coumarins, **(B)** The liquid chromatogram of the standards, **(C)** The liquid chromatogram of test sample. Numbers one to five depict peucedanocoumarin I, praeruptorin A, peucedanocoumarin II, praeruptorin B, and praeruptorin E, respectively.

**FIGURE 4:**
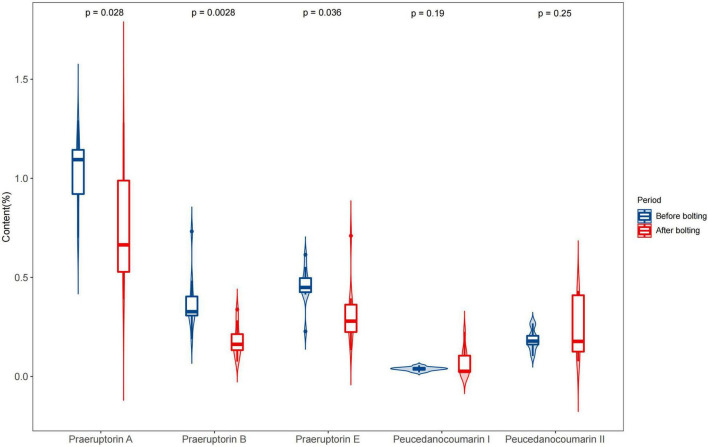
Determination of five coumarins of the unbolted and bolted *P. praeruptorum*. Ten batches of experiments were repeated for each group. The content of each coumarin was calculated as the mean plus standard error. A significant analysis was carried out on the content of coumarins in unbolted and bolted *P. praeruptorum*. “*p*-value < 0.05” means there is a significant difference.

The standards and test samples were determined under the defined chromatographic conditions (Figure 3). Except for praeruptorin A and peucedanocoumarin II, the peaks of peucedanocoumarin I, praeruptorin B, and praeruptorin E had better shape and resolution. The five standards used in the experiment had a common hexacyclic skeleton. However, praeruptorin A and peucedanocoumarin II are isomers and their polarities are closely related to each other, which results in poor separation on a C18 column (Wang et al., 2015). Using the optimized chromatographic method, 10 batches of samples were used to determine the five coumarins (Figure 4).

In the bolted *P. praeruptorum*, the average contents of praeruptorin A, praeruptorin B, and praeruptorin E declined by 26.6, 52.1, and 30.3%, respectively. However, the contents of peucedanocoumarin I and peucedanocoumarin II were raised by 45.7 and 24.8%, respectively. Previous study also demonstrated that the contents of praeruptorin A and praeruptorin B decreased after bolting (Sarkhail et al., 2013). Praeruptorin A and peucedanocoumarin II accumulate in a distinct way depending on the differential expression of their biosynthetic genes, such as *prenyltransferase* (*PT*), *acetyl-CoA acetyltransferase* (*AACT*), and *O-methyltransferase* (*O-MT*) (Song et al., 2014). The major difference between praeruptorin A and praeruptorin B was that the R_2_ group is linked to acetyloxy or angeloyloxy groups, and praeruptorin B has two angeloyloxy groups (Figure 3). However, the content of praeruptorin B was significantly lower than that of praeruptorin A after bolting, which suggested that the expression level of *PT* genes was more capable of influencing the biosynthesis of this group of coumarins. In addition, the molecular weight of peucedanocoumarin I had only two hydrogen atoms more than that of praeruptorin A, implying that peucedanocoumarin I was generated from praeruptorin A under the catalysis of some oxidoreductases. The content of peucedanocoumarin I increased, presumably due to the transformation of praeruptorin A. This also explains the decline of praeruptorin A in the bolted *P. praeruptorum*.

### Identification of Coumarin Biosynthetic Genes of Unbolted and Bolted *P. praeruptorum*

**FIGURE 5:**
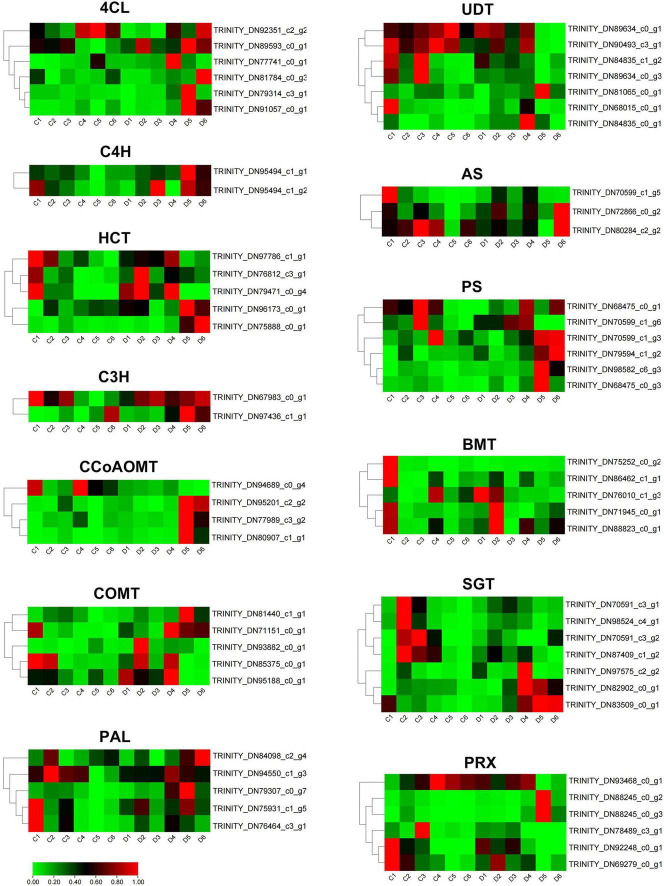
The expression profiles of coumarin biosynthetic genes of the unbolted and bolted *P. praeruptorum*. TPM values were used to compare the gene expression between the two groups. These values were normalized by the log2 function. The red represents the fold change in expression levels reaching two folds, the green represents the fold change in expression levels reaching one fold, and the black represents the fold change between one and two folds.

To further investigate the coumarin biosynthesis of *P. praeruptorum*, we compared and analyzed unbolted and bolted transcriptomic data in the annual plants (Supplementary Table 2). The hierarchical clustering divides these differential genes into two subgroups (Supplementary Figure 1). A total of 1,573 differentially expressed genes were screened out, of which 298 genes were up-regulated and 1,275 genes were down-regulated. Among them, 63 candidates involved in coumarin biosynthesis, including *PAL, 4CL, C4H, C3H, HCT, COMT, CCoAOMT, UDT, AS, PS, BMT, SGT*, and *PRX*, were identified from the transcriptomics data (Supplementary Table 3). Compared with the unbolted, *4CL, C4H, PS2*, and *C3H* were significantly upregulated, whereas *UDT, COMT, AS, BMT*, and *PRX* were remarkably downregulated in the bolted *P. praeruptorum* (Figure 5). The expression levels of some homologs involved in coumarin biosynthesis, such as *PAL, HCT, AS, PS, SGT, etc.*, had emerged different expressions. The reduction of the key genes in the umbelliferone branch pathway may result in the decline of coumarin accumulation. Such genes with bidirectional expression patterns suggest that functional divergence and spatiotemporal expression exist in these paralogs (Siwinska et al., 2018).

### The Expression Profile and *q*PCR Validation of Genes Associated With Coumarin Biosynthesis

**FIGURE 6:**
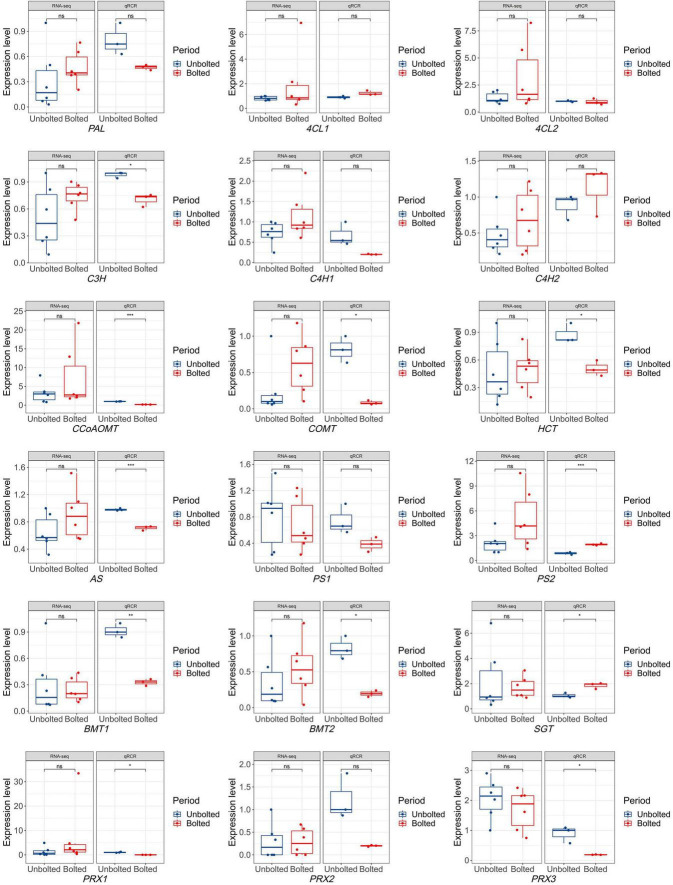
The expression levels of eighteen coumarin biosynthetic genes obtained by RNA-seq and *q*PCR analysis. The expression level obtained by RNA-seq technology contains 6 biological replicates, while the expression level obtained by qPCR method contains 3 biological replicates. Significant differences in the expression levels of coumarin biosynthesis genes were analyzed in the unbolted and bolted *P. praeruptorum*. One asterisk indicates significant difference between two periods. Three asterisks indicate extremely significant difference between two periods. “ns” indicates no significance. *denote there is significant difference between two groups (0.01 < *P* <0.05). **denote there is extremely significant difference between two groups (*P* < 0.01). ***denote there is extremely significant difference between two groups (*P* < 0.001).

To narrow down the biosynthetic genes related to coumarins, eighteen important genes involved in the coumarin biosynthesis pathway were identified from 63 candidates to determine which genes were differentially expressed in the unbolted *P. praeruptorum*. As shown in Supplementary Figure 2, the coumarin biosynthesis pathway is a branch of the phenylpropanoid metabolism. *p*-Coumaric acid is situated at the crossover point of this branch. Through multi-step reactions, *p*-coumaric acid subsequently forms a linear coumarin scepolin under the catalysis of C3H, CA2H, OMT, and SGT. *p*-Coumaric acid is catalyzed by *4*CL, C3H, HCT, COMT, CCoAOMT to form feruloyl-CoA, which will further form G-type lignin monomer. p-Coumaric acid is catalyzed by C’2H and lactionization to yield umbelliferone, the precursor of furano- and dihydropyranocoumarin (Supplementary Figure 2). Compared with the expression profiling of coumarin biosynthetic genes in the bolting stage, the expression levels of almost all genes were not significantly different. Except for *PS1* and *PRX3*, the average expression levels of the other genes were upregulated in the bolted stage. To verify the expression of the candidate genes, we further detected the expression level of the genes by *q*PCR analysis. The expression of the *PAL*, *C4H1*, *C3H*, *HCT*, *COMT*, *CCoAOMT*, *AS*, *PS1*, *BMTs*, and *PRXs* were decreased in the bolted *P. praeruptorum*, whereas the expression of the *4CL1*, *C4H2*, *PS2*, and *SGT* genes were increased (Figure 6). These results were inconsistent with the expression profile of coumarin biosynthesis genes. We re-analyzed the differences between the transcriptome and *q*PCR analysis results. From the measured expression data, it may be that the expressions of the biological replicates of the transcriptome are in a wide range, and the average expression levels of most genes in the bolting stage are not significantly different.

### The Phylogenetic Analysis and Expression Profile of ABC Transporters in *P. praeruptorum*

**FIGURE 7:**
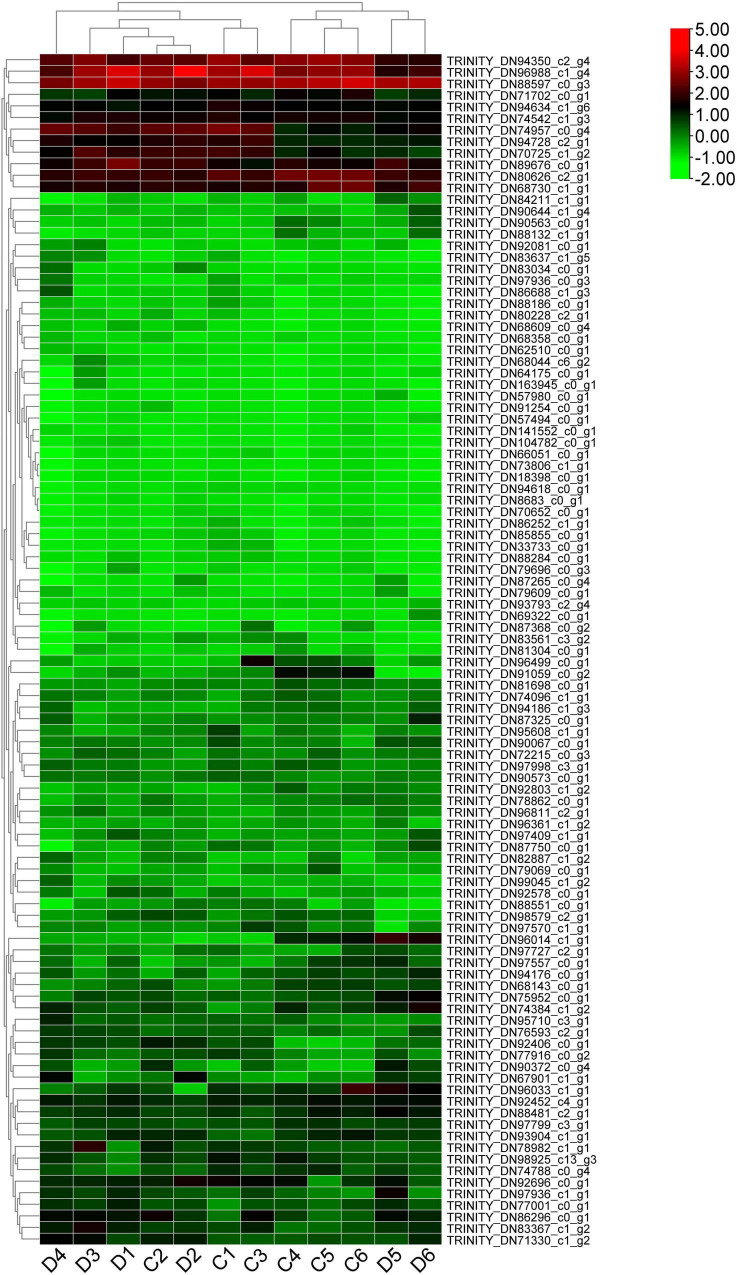
The expression profile of the possible ABC transporter genes in the unbolted and bolted *P. praeruptorum*. The TPM values are normalized by the log2 function. The expression patterns of *PpABC* genes can be divided into four groups: the first group has higher levels of expression both at the bolted and unbolted stages. The second group displayed high expressions prior to bolting and low expressions after bolting. The third group is the absolute opposite of the second group. The fourth group had lower expression both at the bolted and unbolted stages.

ATP-binding cassette (ABC) transporters are a wide and ancestral transmembrane protein family found in many natural species, which have received a lot of attention because of their multiple biological functions (Caña-Bozada et al., 2019; Huang et al., 2021). Here, 188 tentative ABC transporter genes or fragments were screened out from the transcriptome data (Supplementary Table 4). By constructing a phylogenetic tree with 129 ABC transporters from *A. thaliala*, only a limited number of transcripts could be clustered with the ABC family genes from *P. praeruptorum* (Supplementary Figure 3). It was conceivable that these genes or segments included only one or a few domains inside the transmembrane domains (TMDs) and nucleotide-binding domains (NBDs) domains and had little homology with the Arabidopsis ABC transporter family. Most of the *PpABC* genes have high homology with the *AtABCA/ABCD/ABCE/ABCG/ABCI* subfamilies. TRINITY_DN96014_c1_g1 shares a high homology with *AtABCG22*. *TRINITY DN96811_c2_g1* and *TRINITY_DN96361_c1_g2* have a high homology with *AtABCG37*. *TRINITY_DN94634_c1_g6*, *TRINITY_DN78862_c0_g1*, and *TRINITY_DN181606_c0_g1* all have high homology with the *AtABCG2* family. *TRINITY_DN63312_c0_g1* has high homology with *AtABCC10*. *TRINITY_DN80626_c2_g1*, *TRINITY_DN102046_c0_g1* have high homology with *ABCA* family. *TRINITY_DN82877_c1_g2* has high homology with *AtABCB5*. We further analyzed the expression profile of 103 *PpABC* genes (Supplementary Table 5). The results indicated that the expression levels of these genes had several patterns. The first group of genes had higher expression in both unbolted and bolted *P. praeruptorum*, such as *TRINITY_DN94350_c2_g4*, *TRINITY_DN96988_c1_g4* and *TRINITY_DN88597_c0_g3*. *TRINITY_DN94350_c2_g4* shared more homology with *AtABCE2*, whereas the other two genes shared more with *AtABCF1*. The second group of genes had low expression in the unbolted *P. praeruptorum* and increased expression in the bolted, such as *TRINITY_DN86296_c0_g1*, *TRINITY_DN749574350_c0_g4*, and *TRINITY_DN94728_c2_g1*. *TRINITY_DN86296_c0_g1* and *TRINITY_DN7495_c0_g4* were both classified as members of the *AtABCG* subfamily, and *TRINITY_DN94728_c2_g1* was found to be related to *AtABCB20*. The third group of genes had higher expression in the unbolted *P. praeruptorum* and lower expression in the bolted, such as *TRINITY_DN96014_c1_g1* and *TRINITY_DN91059_c0_g2*. *TRINITY_DN96014_c1_g1* and *TRINITY_DN91059_c0_g2* shared more homology with *AtABCG22* and *AtABCG4*, respectively. The remaining genes were expressed at lower levels both in unbolted and bolted *P. praeruptorum* (Figure 7). ABC transporters play multiple roles in trafficking ions, carbohydrates, lipids, xenobiotics, antibiotics, medicines, and heavy metals (Gadsby et al., 2006; Khan et al., 2020). *AtABCB1* and *AtABCB2* are known as auxin transporters. Overexpression of *AtABCB1* promoted hypocotyl cell elongation (Lewis et al., 2007). *A. thaliala*, *Z. mays*, and *O. sativa* also contain *ABCC* subfamily members that are responsible for phytate trafficking (Nagy et al., 2009; Badone et al., 2010; Tagashira et al., 2015). Both *AtABCC1* and *AtABCC2* mediate tolerance to cadmium (Cd) and mercury via vacuolar sequestration (Park et al., 2012). *AtABCF3* was involved in root development and growth (Kato et al., 2009). *ABCG* subfamily members have been associated with cuticle development and mental impairment (Bessire et al., 2011; Fourcroy et al., 2014; Lefèvre et al., 2018). *AtABCG36* stimulated Cd uptake in root epidermal cells and was induced by Cd treatment (Kim et al., 2007). *AtABCG37* was primarily responsible for delivering highly oxygenated coumarins to root exudation (Ziegler et al., 2017). The *ABCG* subfamily might be the main regulators participating in the extracellular secretion and transport of coumarin.

### The Interaction Network of Coumarin Biosynthetic Genes

To clarify which genes were involved in the regulation of coumarin biosynthesis, the WGCNA data from the previous study was deeply mined (Song et al., 2021). Among thirteen gene modules, three modules relevant to coumarin biosynthesis were screened out by a gene-module correlation analysis (Supplementary Table 6). A total of 210 gene pairs were associated with the genes related to coumarins biosynthesis (Supplementary Table 7). *TRINITY_DN77989_c3_g2* (*CCoAOMT*), *TRINITY_DN68475_c0_g1* (*PS1*), *TRINITY_ DN95494_c1_g1* (*C4H1*), *TRINITY_DN79594_c1_g2* (*PS2*), and *TRINITY_DN90493_c3_g1* (*UDT*) interacted strongly with some genes in the turquoise module. *PS1* and *C4H1* have strong interactions with *TRINITY_DN97736_c4_g6* (*UPL3*), implying that *UPL3* is involved in the ubiquitination of the two proteins. *TRINITY_DN77619_c2_g1* (*HSL1*) interacts with *UDT*. *TRINITY_DN74096_c0_g1* (*ABCB1*) interacts strongly with *TRINITY_DN98338_c2_g1* (*LOX2*), *TRINITY_DN95583_c1_g4* (*MPK3*), and *TRINITY_DN79029_c0_g2* (*Scarecrow-like TF*). *TRINITY_DN75913_c1_g5* (*PAL*) interacts with *TRINITY_DN84719_c0_g2* (*aquaporin 1*) and *TRINITY_DN92098_c0_g7* (*EARLI 1*) in the yellow-green module (Supplementary Figure 4).

## Conclusion

Early bolting seriously affects the yield and quality of *P. praeruptorum*. The underlying mechanisms of the ineffectiveness of the bolted *P. praeruptorum* for medicinal purposes remain unclear. We found that lignification was more severe in the root of bolted *P. praeruptorum*. The contents of praeruptorin A, B, and E were lower in the bolted *P. praeruptorum*. We further compared the transcriptome at each bolting stage and found that some genes on the phenylpropanoid pathway branch were involved in coumarin biosynthesis, such as *PAL*, *C4H*, *HCT*, *COMT*, and *CCoAOMT*. Additionally, we screened several ABC transporters implicated in coumarin transport, including those belonging to the *ABCA/D/E/G/I* subfamilies. The *ABCG* subfamily may also play a role in the transportation of coumarin during the bolting stage. The network of co-expressed genes indicated that *PS1* and *C4H1* both have strong interactions with *UPL3*. Bolting may have a negative effect on the accumulation of coumarins in *P. praeruptorum* and the regulation of associated biosynthetic genes. Our results provide some scientific references for the quality evaluation of *P. praeruptorum* drugs. However, more evidence is needed to figure out how bolting changes the endogenous signaling cascades and how this influences the activation of downstream genes.

## Data Availability Statement

The datasets presented in this study can be found in online repositories. The names of the repository/repositories and accession number(s) can be found in the article/Supplementary Material.

## Author Contributions

BH and CS discussed the writing plan. CS, XL, and BJ drafted the manuscript. CS, XL, LL, PW, MM, CC, and FW edited the manuscript. XL, BJ, BL, and GW conduct the experiment. BH acquired the funding. All authors have read, reviewed, and approved the submitted version.

## Conflict of Interest

The authors declare that the research was conducted in the absence of any commercial or financial relationships that could be construed as a potential conflict of interest.

## Publisher’s Note

All claims expressed in this article are solely those of the authors and do not necessarily represent those of their affiliated organizations, or those of the publisher, the editors and the reviewers. Any product that may be evaluated in this article, or claim that may be made by its manufacturer, is not guaranteed or endorsed by the publisher.

## Footnotes

1
https://www.ncbi.nlm.nih.gov/sra

2
http://transdecoder.github.io/
