# Supplementary figures and images for: Comparative Transcriptomics Unveil the Crucial Genes Involved in Coumarin Biosynthesis in Peucedanum praeruptorum Dunn
Source: Front Plant Sci. 2022 May 17;13:899819. doi: 10.3389/fpls.2022.899819 (PMC9152428; doi:10.3389/fpls.2022.899819)

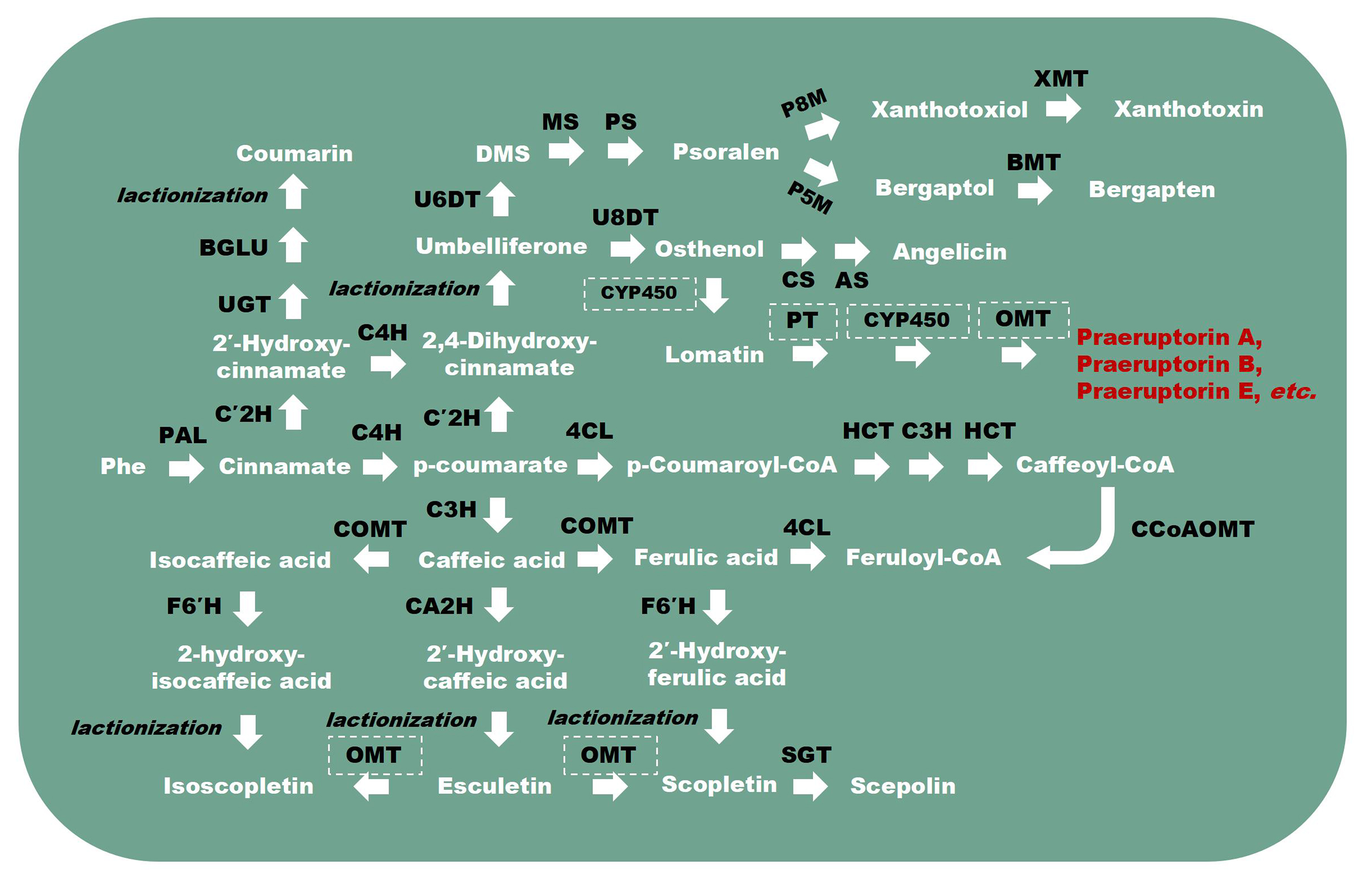

Supplement: Supplementary file 2 [file Image_1.JPEG]

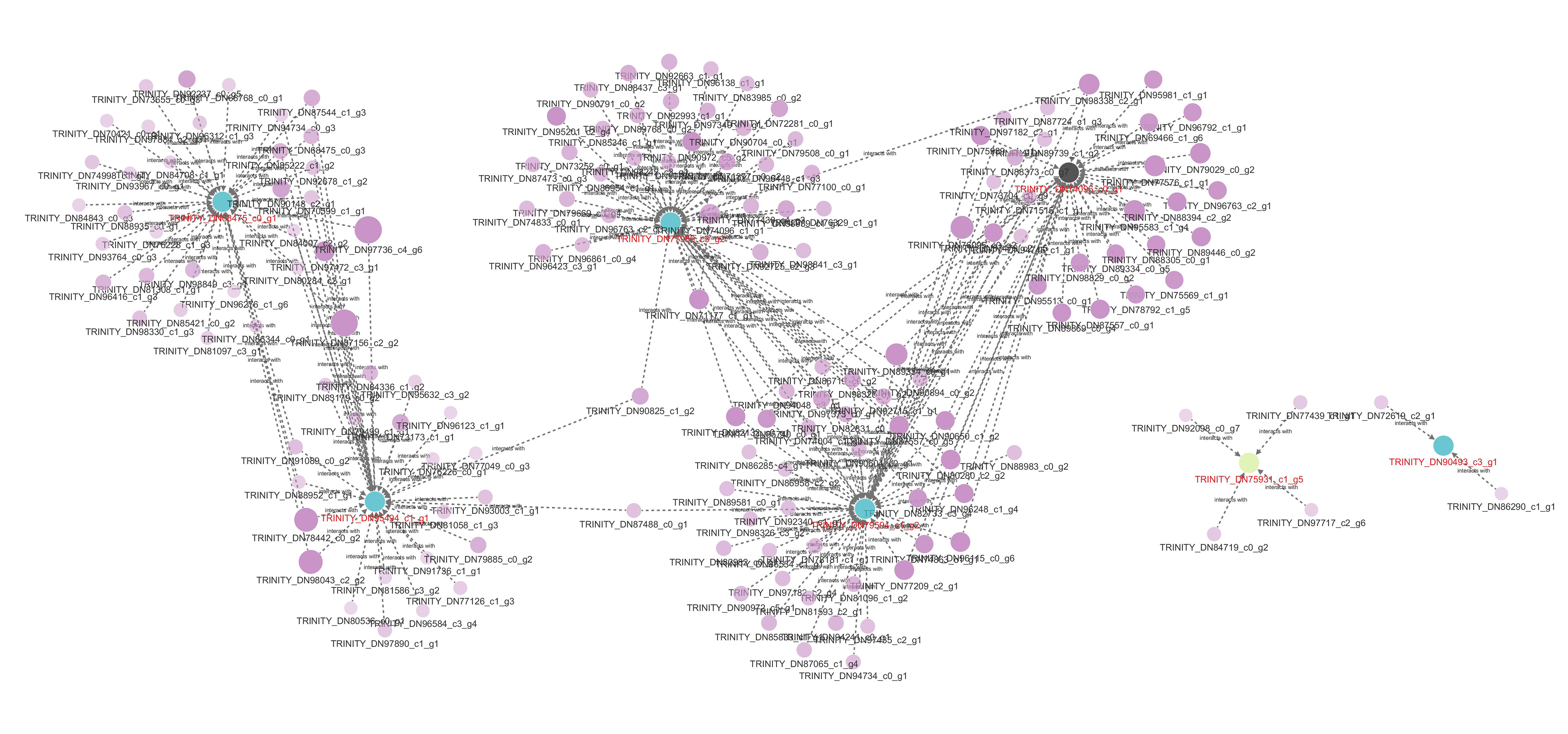

Supplement: Supplementary file 4 [file Image_3.JPEG]
